# Obesity, Xenobiotic Intake and Antimicrobial-Resistance Genes in the Human Gastrointestinal Tract: A Comparative Study of Eutrophic, Overweight and Obese Individuals

**DOI:** 10.3390/genes10050349

**Published:** 2019-05-07

**Authors:** Marjorie Raquel Anariba Sarmiento, Thais Oliveira de Paula, Francis Moreira Borges, Alessandra Barbosa Ferreira-Machado, Juliana Alves Resende, Ana Paula Boroni Moreira, Sheila Cristina Potente Dutra Luquetti, Dioneia Evangelista Cesar, Vânia Lúcia da Silva, Claudio Galuppo Diniz

**Affiliations:** 1 Laboratory of Bacterial Physiology and Molecular Genetics, Department of Parasitology, Microbiology and Immunology, Federal University of Juiz de Fora, 36036-330 Juiz de Fora, MG, Brazil; marjorieanariba@gmail.com (M.R.A.S.); tata84farm@yahoo.com.br (T.O.d.P.); francismborges@gmail.com (F.M.B.); alessandrabferreira@yahoo.com.br (A.B.F.-M.); vanocabr@gmail.com (V.L.d.S.); 2 Department of Pharmacy and Nutrition, Federal University of Espirito Santo, 29500-000 Alegre, ES, Brasil; ju.alves.resende@gmail.com; 3 Department of Nutrition, Federal University of Juiz de Fora, 36036-330 Juiz de Fora, MG, Brazil; apboroni@yahoo.com.br (A.P.B.M.); scpotentedutra@yahoo.com.br (S.C.P.D.L.); 4 Department of Biology, Federal University of Juiz de Fora, 36036-330 Juiz de Fora, MG, Brazil; dioneia.cesar@gmail.com

**Keywords:** microbiota, dysbiosis, xenobiotic intake, obesity, antimicrobial resistance

## Abstract

Although lifestyle and physiology in obese individuals are accepted to lead to changes in the intestinal microbiota, uncertainty remains about microbiota dysbiosis, and xenobiotics intake, as a source of selective pressure, independent of antimicrobial chemotherapy. The aim of this study was to compare the occurrence of antimicrobial resistance genetic markers (ARG) in faecal specimens of eutrophic, overweight and obese individuals, and their correlation with xenobiotic intake and gut bacteria density. *Methods*: This was a cross-sectional case-controlled study including 72 adult participants with no record of intestinal or systemic diseases, or recent use of antimicrobials, grouped as eutrophic, overweight, or obese. Anthropometric profile, eating habits and oral xenobiotics intake were recorded. Faecal metagenomic DNA was used to screen for ARG by PCR, and to measure bacterial groups by fluorescence in situ hybridization (FISH). Student’s *t* and Wilcoxon tests were used to compare means and differences in ARG detection (95% confidence intervals). Correlation analyses (odds ratio) and relationships between bacteria density and ARG were determined. *Results*: Increase in abdominal circumference, waist circumference, hip, waist-hip ratio, BMI, carbohydrate, fibres, and total calorie intakes were different from eutrophic to obese participants. Habitual use of antihypertensive and anti-inflammatory drugs, antacids, and artificial sweeteners were associated mainly with obesity and overweight. Nutritional supplements were associated to the eutrophic group. ARG screening showed differences being more frequent among obese, and positive for 27 genetic markers related to β-lactams, tetracyclines, the macrolide lincosamide and streptogramin group, quinolones, sulfonamides, aminoglycosides, and efflux pump. Positive correlation between ARG and BMI, caloric intake, and intake of xenobiotics, was observed for obese individuals. Relationships among ARG detection and bacteria densities were also different. *Conclusions*: This study reinforces the hypothesis that obese individuals may harbour an altered gut microbiota, if compared to eutrophic. The overweight individuals display a transitional gut microbiota which seems to be between eutrophic and obese. Furthermore, the increased xenobiotic intake associated to obesity may play an important role in the antimicrobial resistance phenomenon.

## 1. Introduction

The functionality of the human body depends on its dynamic ecological balance with resident microbiota, which create essential physiological characteristics for an individual’s survival [1,2]. In humans and other warm-blooded animals, the microbiota associated with the gastrointestinal tract is responsible for several fundamental functions in host physiology, including processing of non-digestible dietary components, development, and maturation of the immune system, and processing of xenobiotics [1,3,4].

It is accepted that gastrointestinal tract also provides a large reservoir of antibiotic resistance genes through the microbiota, defined as the intestinal resistome. This includes genes of clinical relevance defined, in turn, as the clinical resistome [5]. However, little is known about the occurrence of such genetic markers associated to the human microbiota at different anatomical sites in subjects without recent antibiotic exposure. It is suggested that microbiota resilience is associated with post-antibiotic equilibrium states [6]. The intestinal resistome is established 1–2 months after birth, and its enrichment is associated with factors related to the child’s environment [7]. Resistance determinants in the gut have also been documented in the microbiota of isolated indigenous populations that have never received antibiotic treatment, and they are not related with the synthesis of secondary metabolites, that might suggest they are constitutively expressed [8,9]. 

With regard to gut microbiota dysbiosis, obesity has been declared a pandemic by the World Health Organization (WHO) and its close relationship with the intestinal microbiota has been extensively described [10,11,12,13]. Different factors associated with nutrition, physiological status and lifestyle in obese individuals are accepted to lead to intense changes in the microbiota and its metabolites in the intestinal ecosystem [14]. In addition, ingestion of therapeutic agents and exposure to environmental pollutants in food and water are among the main routes of xenobiotic exposure which may push the intestinal microbiota towards dysbiosis, potentially acting as selective pressure factors in the gut environment [15]. 

The world is faced with an emerging and uncontrolled increase in antimicrobial resistance. However, there are no reports on the role of obesity and its associated gut microbiota dysbiosis and xenobiotics intake, as a source of selective pressure, independent of antimicrobial drug ingestion. This study compared the occurrence of antimicrobial resistance genetic markers (ARG) related to the human clinical resistome in faecal specimens of eutrophic, overweight and obese individuals, and their correlation with representative bacterial groups commonly thought to carry these genes.

## 2. Subjects and Methods

### 2.1. Participants, Dietary Assessment, and Specimen Collection 

Adult participants (n = 72) were selected from the community and from a nutrition clinic at the Federal University of Juiz de Fora (UFJF) teaching hospital (Juiz de Fora, MG, Brazil). Selection criteria for participants in this study included age between 18 and 60 years, no record of intestinal diseases, no diabetes, and no record of antimicrobial drugs use during the last month. Anthropometric measurements were taken after written informed consent. This was a cross-sectional and descriptive study approved by the Human Research Ethics Committee (479.002/2013) at UFJF. 

A quantitative food frequency questionnaire (QFFQ) was used to estimate the habitual dietary intake of the participants and further evaluate their macronutrients ingestion (carbohydrates, proteins, fibre, and lipids). For each QFFQ item, the volunteers reported the average frequency of consumption (daily, weekly, or monthly) for the last six months, and the size of the portion ingested. The analysis of habitual food consumption was performed using tables of food composition [16]. The participants provided information on oral intake of xenobiotics, such as prescription or non-prescription drugs, hormones, nutritional supplements and artificial sweeteners. The participants were grouped as eutrophic, overweight, or obese in accordance with their body mass index (BMI). 

Faecal samples were collected in sterile vials given to the participants after obtaining informed consent and physical data measurements. All the participants were instructed to collect the faecal samples in the next morning after ablutions and send them immediately to the laboratory for analysis.

### 2.2. DNA Extraction 

The faecal samples were homogenized and 0.2 g aliquots were submitted to metagenomic DNA extraction using the QIAamp DNA Stool Mini Kit, and the automated QIAcube robotic workstation (Qiagen, Hilden, NRW, Germany). Samples were processed according to the manufacturer’s instructions with the following modifications. Three glass beads were added per sample at the lysis step, and homogenized by vortexing for two minutes. Then, the samples were incubated at 95 ° C for 15 minutes and subsequently vortexed vigorously for two additional minutes. After this step, 300 µL of the buffer was added to the faecal suspensions, followed by mixing, incubation and centrifugation. 

DNA concentration was determined by fluorimetry using the QubitTM 2.0 fluorimeter, with the Qubit dsDNA HS Assay Kit (Life Technologies, Carlsbad, CA, USA), and integrity was assessed by 0.8% agarose gel electrophoresis. The resulting purified DNA was stored at −70 °C.

### 2.3. Screening of ARG by PCR

A set of 55 ARG of human clinical relevance (clinical resistome), including genetic markers related to efflux pumps, were used to screen faecalmetagenomic DNA by conventional PCR, using specific primers and amplification conditions described previously (Table S1, Supplementary Data). PCR products were analysed by 1.5% (*w*/*v*) agarose gel electrophoresis in 1xTBE buffer. DNA bands were visualized with ethidium bromide on an ultraviolet light transilluminator. Amplicons sizes were estimated by comparison with a co-electrophoresed 100 bp DNA ladder (Ludwig Biotec, Alvorada, RS, Brazil). 

Negative controls were performed as reactions without template DNA. As verification of their origin, amplicons were purified using the QIAquick Gel Extraction Kit (Qiagen) and the DNA fragments obtained were sequenced in an ABI Prism 3730 DNA sequencer (Applied Biosystems, Foster City, CA, USA). Sequences were analysed with the Basic Local Alignment Search Tool (BLAST) against the nucleotide database of the National Centre for Biotechnology Information (NCBI).

### 2.4. Screening of Bacterial Population by Fluorescence in Situ Hybridization (FISH)

Paraformaldehyde (20% (*w*/*v*)) in phosphate-buffered saline (PBS) was added to 0.2 g aliquots of the faecal samples to a final concentration of 2%. Tween 80 (0.001% *v*/*v*) was then added and the mixture was incubated at room temperature for 60 min. The samples were sonicated on ice at amplitude of 90%, for 3 × 60 s pulses with 60 s of resting time between each pulse. Then, the bacterial cells were concentrated on polycarbonate filters (25 mm diameter, pore size 0.22 µm; Whatman VWR, Mississauga, ON, Canada), after centrifugation for 5 min at room temperature. The collected cells were washed twice with 5 mL of PBS. 

The polycarbonate filters were subjected to the fluorescence in situ hybridization (FISH) protocol, according to Cottrell and Kirchman [17], where RNA-targeted oligonucleotide probes were used to estimate the intestinal bacterial composition. The filters were placed on glass slides and covered with 40 μL of hybridization solution containing the probes at a final concentration of 2.5 ng/μL. Hybridization solution contained 0.9 M NaCl, 20 mM Tris-HCl pH 7.2, 5 mM EDTA, 0.01% SDS, and variable concentrations of formamide. The probe sequences and hybridization conditions are given in Table S2, Supplementary Data. Incubations were performed in a hybridization chamber at 42 °C overnight. After hybridization, filters were transferred to pre-warmed washing solution (20 mM Tris-HCl pH 7.4, 5 mM EDTA, 0.01% SDS, and NaCl at a concentration dictated by the probe) and incubated at 48 °C for 15 minutes. Filters were labelled with 100 μL of 4′,6-diamidino-2-phenylindole (DAPI), at a final concentration of 2 mg/mL, for three minutes at room temperature, and then washed three times with 80% ethanol, dried on paper and mounted on glass slides using a glycerol:PBS (7:3) solution.

Bacterial cells on the filter sections were observed using a BX60 microscope (Olympus, Tokyo, Japan). Microbes were analysed in 10 random fields for each probe from each sample. For each microscopic field, two metrics were determined, the total number of DAPI-stained cells and the number of cells stained with the specific probe. All counts were corrected by subtracting those obtained with the negative control probe. From all the microscopic fields analysed bacterial density was calculated. The experiments were performed in duplicate.

### 2.5. Statistical Analysis

Student’s *t* test was performed to compare means, and the Wilcoxon test was used to determine if there were significant differences in the frequency of detection of the resistance markers. Correlation analysis between the occurrence of resistance markers, and anthropometric and nutritional characteristics were assessed by calculating the odds ratio (OR) [18]. An OR value of <1.0 indicated a negative correlation, that is, the probability is lower in the first group than in the second or the condition under study is equally likely in both groups. An OR value equal to 1.0 indicated absence correlation and OR value >1.0 indicated a positive correlation. Overall, confidence intervals were set at 95%. Relationships between density of the bacterial groups and the frequency of resistance markers detected were evaluated using a ratio between the absolute number of the resistance markers and the relative frequency of each bacterial group.

## 3. Results 

The 72 adult volunteers selected from the community and from a nutrition clinic at the UFJF teaching hospital were divided into 3 groups of 24 individuals, classified as eutrophic (five male and 19 female), overweight (12 male and 12 female) and obese (nine male and 15 female). In total, 61.11% were female and 38.89% were male, with an average age of 39.61 years old (eutrophic 37.91 ± 12, overweight 38.12 ± 13.38, and obese 42.79 ± 11.67). Regarding their anthropometric characteristics, there was a gradual increase in the abdominal circumference, waist circumference, hip, waist-hip ratio, and body mass index from eutrophic to obese participants. The average values for anthropometric measurements (BMI, abdominal circumference, waist circumference, hip circumference and waist-hip ratio) were of statistical significance (*p* < 0.05) between eutrophic, overweight, and obese volunteers, considering both male and female participants. (Table S3, Supplementary Data). Considering diet characteristics, although the mean daily lipids intake did not differ between the evaluated groups based on the QFFQ, the overall daily carbohydrate and fibre intakes were significantly different between eutrophic and obese groups (*p* < 0.05). The overall mean daily calorie (kcal) intake was also significantly different between eutrophic and obese participants (*p*< 0.05; Table S3, Supplementary Data).

Although antimicrobial use over the past 30 days was an exclusion criterion in this study, participants (mainly within the obese group) declared eventual or habitual oral intake of xenobiotics such as prescription or non-prescription drugs, hormones, nutritional supplements and artificial sweeteners. Among obese participants, the use of antihypertensive medication was reported by 79.2%, with antacids and anti-inflammatories being habitually taken by nearly 10% of this group. Only 1 obese participant declared a regular intake of diuretics, barbiturates or antidepressants (Table S4, Supplementary Data). Regular intake of antihypertensive was also declared by 12.5% of overweight participants. For eutrophic group, 21% of the participants declared a habitual intake of nutritional supplements such as whey protein, glucosamine and chondroitin sulphate. Habitual use of artificial sweeteners was reported by all groups, but their use was mainly associated with obese (41.6%) and overweight individuals (21%). These xenobiotics were grouped according to their chemical composition. 

**Figure 1:**
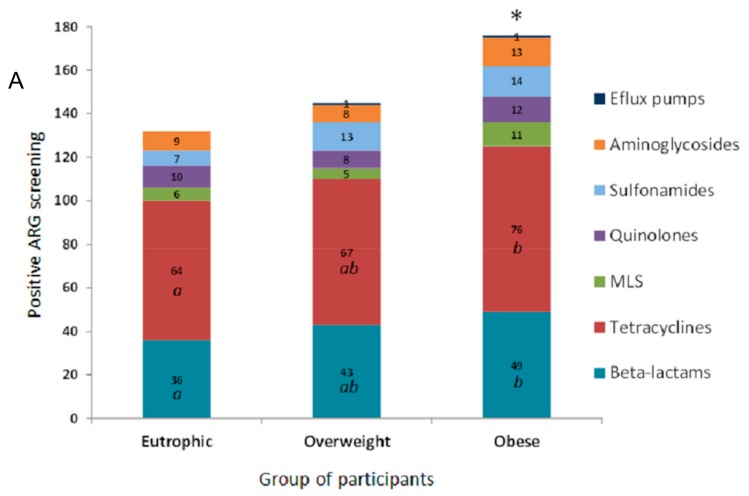

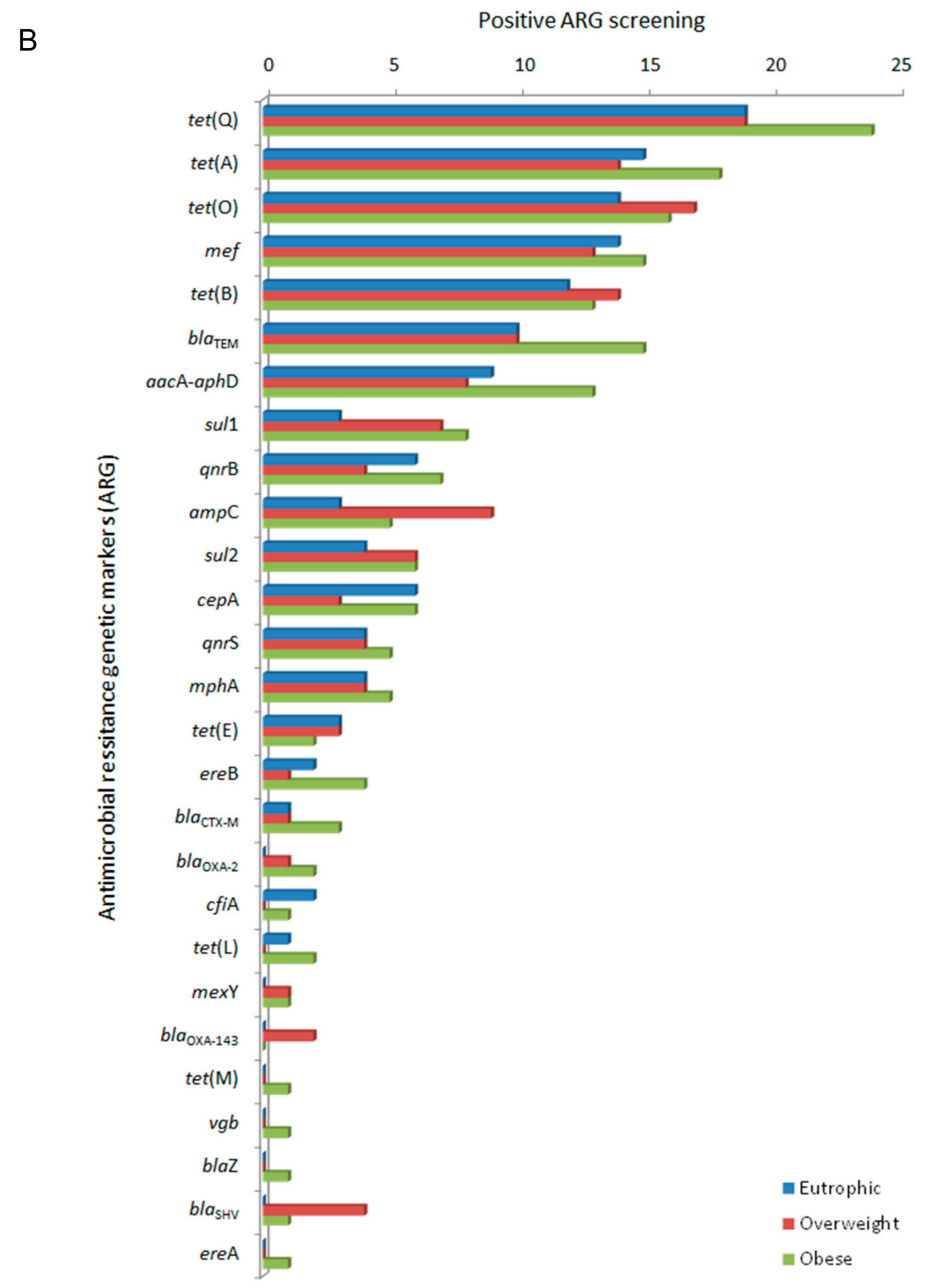
Distribution of antimicrobial resistance genetic markers (ARG) related to different classes of antimicrobial drugs accordingly to positive screening by PCR of the faecal metagenome from eutrophic, overweight, and obese individuals. (**A**) ARG were clustered based on their chemical structure or phenotype, such as β-lactams, tetracyclines, macrolide lincosamide and streptogramin group (MLS), quinolones, sulfonamides and aminoglycosides, and efflux pumps. For the obese, overweight and eutrophic groups, ARG screening was positive in 176, 145 and 132 tests, respectively. Captions *a*, *b*, and * indicate statistically significance (*p* < 0.05). (**B**) Absolute distribution (number of positive screenings) of 27 positive ARG out of 55 tested genetic markers in intestinal metagenomes from eutrophic, overweight and obese individuals. β-lactams (*amp*C, *bla*_CTX-M_, *bla*_TEM_, *bla*_SHV_, *bla*_OXA-2_, *bla*_OXA-143_, *cfi*A, *cep*A, *bla*Z, *mef*); tetracyclines (*tet*(A), *tet*(B), *tet*(E), *tet*(L), *tet*(M), *tet*(O), *tet*(Q)); macrolides, lincosamide and streptogramin group (MLS) (*mph*A, *ere*A, *ere*B, *vgb*); quinolones (*qnr*B, *qnr*S); sulfonamides (*sul*1, *sul*2); aminoglycosides (*aacA-aphD*); and efflux pumps (*mex*Y).

In this study, the evaluated clinical resistome comprised the screening of 55 ARG. The ARG were representative of antimicrobials commonly used in both human and veterinary medicine. ARG screening was positive for 27 genetic markers related to different classes of antimicrobial drugs based on their chemical structure, such as β-lactams, tetracyclines, the macrolide lincosamide and streptogramin group (MLS), quinolones, sulfonamides, and aminoglycosides. The efflux pump related to *mex*Y, was also detected (Figure 1A). The clinical resistome representative of the gastrointestinal tract was significantly different between the three groups of individuals, especially considering the detection rates of ARG in the obese group. Genetic markers related to tetracycline resistance were the most frequently observed in all groups, followed by β-lactams, aminoglycosides, sulfonamides and quinolones. In general, ARG were detected at higher frequencies among obese individuals (Figure 1B).

**Figure 2:**
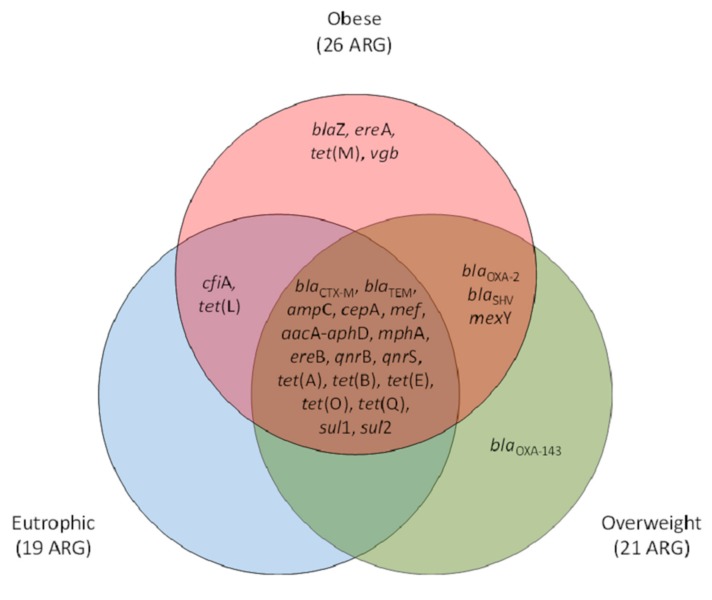
Venn diagram representative of the antimicrobial resistance genetic markers (ARG) occurrence and qualitative clustering according to their positive screening exclusively or shared between eutrophic, overweight, and obese individuals. β-lactams (*amp*C, *bla*_CTX-M_, *bla*_TEM_, *bla*_SHV_, *bla*_OXA-2_, *bla*_OXA-143_, *cfi*A, *cep*A, *bla*Z, *mef*); tetracyclines (*tet*(A), *tet*(B), *tet*(E), *tet*(L), *tet*(M), *tet*(O), *tet*(Q)); macrolides, lincosamide and streptogramin group (MLS) (*mph*A,, *ere*A, *ere*B, *vgb*); quinolones (*qnr*B, *qnr*S); sulfonamides (*sul*1, *sul*2); aminoglycosides (*aacA-aphD*); and efflux pumps (*mex*Y).

For the three groups of participants (eutrophic, overweight, and obese), a core set of 17 ARG was observed. Exclusive ARG were observed within obese (n = 4) and overweight (n = 1) clinical resistomes. Obese and overweight or obese and eutrophic shared three (*bla*_OXA-2_, *bla*_SHV_, *mex*Y) and 2 (*cfi*A, *tet*(L)) of the screened ARG, respectively. In total, 26 ARG were observed for obese individuals, whereas 21 and 19 were observed for those overweight and of eutrophic, respectively. The genetic marker *bla*_OXA-143_ was observed exclusively within overweight individuals, and *blaZ*, *ere*A, *tet*(M), and *vgb* were found exclusive within the obese group (Figure 2).

**Figure 3:**
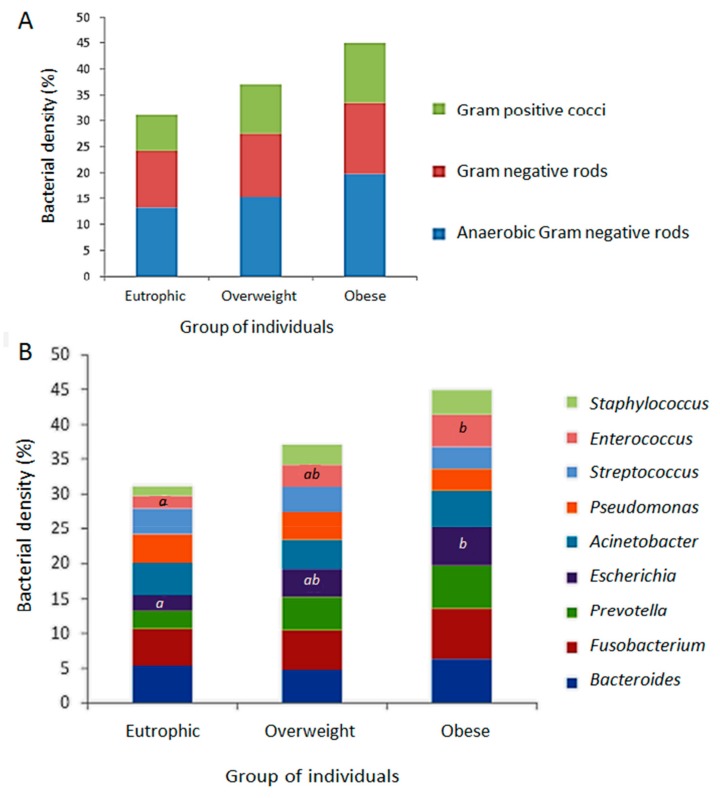
Relative density of the bacterial groups associated to the positive screening of antimicrobial resistance genetic markers (ARG), determined by fluorescence in situ hybridization (FISH) from the faecal metagenomes of eutrophic, overweight and obese individuals. (**A**) Quantitative evaluation by clustering different bacterial groups according to the hybridization probes available and relevance of microorganisms: anaerobic Gram-negative rods (*Fusobacterium* spp., *Prevotella*spp., *Bacteroides* spp.); Gram-negative rods (*E. coli*, *Acinetobacter* spp., *Pseudomonas* spp.); Gram-positive cocci (*Staphylococcus* spp., *Enterococcus* spp., *Streptococcus* spp.). (**B**) Distribution of evaluated bacterial groups among eutrophic overweight and obese individuals. Captions *a*, *b*, and * indicate statistically significant results (*p* < 0.05).

FISH was performed to analyse the relative density of the bacterial groups associated to the ARG screened in this study as representative of the gastrointestinal tract clinical resistome. The results allowed a quantitative evaluation, clustering different bacterial groups according to the hybridization probes available and the relevance of microorganisms: (i) anaerobic Gram-negative rods (*Fusobacterium* spp., *Prevotella* spp., *Bacteroides* spp.); (ii) other Gram-negative bacilli (*Escherichia coli*, *Acinetobacter* spp., *Pseudomonas* spp.); and (iii) Gram-positive cocci (*Staphylococcus* spp., *Enterococcus* spp., *Streptococcus* spp.). As expected, Gram-negative anaerobes predominated, followed by Gram-negative rods and Gram-positive cocci. By using the FISH approach, a tendency to higher bacterial density was observed in the obese faecal microbiota followed by those of the overweight and eutrophic, with no significant difference (Figure 3A). The bacterial groups were differently distributed and indicated a higher density of *Fusobacterium* among obese individuals, with significant differences in the quantification of *Enterococcus* and *E. coli* between the eutrophic and obese groups. There were no significant differences between the weight classes for the other bacterial groups (Figure 3B). 

Odds ratio was calculated between the ARG and BMI, caloric intake, use of xenobiotics and artificial sweeteners. It was established as a cut-off parameter that at least 5 ARG corresponded to approximately 10% of all markers assessed. There was a positive correlation between the detection of ARG in the faecal metagenome of obese individuals and all the evaluated parameters (Table 1).

To understand the relationships between density of the bacterial groups and the frequency of ARG detection, ratios between the absolute number of ARG more likely to occur in the different bacterial groups and the relative frequency of each group were determined. The results might suggest that while anaerobic Gram-negative rods and Gram-positive cocci were related to the ARG observed in the faecal metagenome for the eutrophic group, for obese individuals the observed ARG might be related to other Gram-negative bacilli (Table 2).

## 4. Discussion

Obesity is a chronic disease whose complications and metabolic implications are considered among the main health challenges to be addressed in the twenty-first century, along with the increasing antimicrobial resistance phenomenon [19,20]. The disease might be considered a multifactorial metabolic disorder with the participation of hormones, neuropeptides, cytokines, and also the gut microbiota [21,22,23]. 

In this study, participants recruited in Southeastern Brazil and grouped as eutrophic, overweight, and obese, based on their BMI. Nutritional and anthropometric characteristics showed feeding behaviours anticipated for this geographical region [24]. However, obese individuals had a significantly increased consumption of calories, carbohydrates, and fibres if compared to the eutrophic group. According to the literature, a diet rich in sugars might be related to the behaviours of western society and has been associated to a positive energy balance, low-grade inflammation and changes in intestinal permeability leading to obesity [25]. Considering the habitual consumption of proteins and lipids, according to answers given by participants in the QFFQ there was no significant difference between the groups. According to the literature issues related to accuracy might sub-estimate habitual consumption of nutrients, especially regarding obese and overweight participants when answering the QFFQ [26]. 

Notably, obese patients were distinguished overall by the use of antihypertensive, artificial sweeteners, antacids and anti-inflammatories. To date there is no information of how such xenobiotic use might modulate the intestinal microbiota, other than the effects of antimicrobial drugs on the antimicrobial resistance phenomenon [27,28]. 

Regarding modulation of microbial ecosystems, especially intestinal microbiota, by antimicrobial exposure, the literature suggests the presence of subpopulations of resistant microorganisms in the resident microbiota of different mammals, regardless of whether there has been direct or indirect exposure to drugs or other xenobiotics. These observations include studies in native non-antimicrobial consuming populations and native populations inhabiting isolated environments [6,8,9]. 

In this current study, 55 genetic markers were evaluated as representative of the human clinical resistome in the gastrointestinal tract. These ARG are related mainly to antimicrobial drugs routinely used for treatment and prophylaxis of infectious diseases in humans and animals in Brazil. Overall, 27 different ARG related to bacterial resistance to different antimicrobial drugs classes were detected. Of these, 17 were shared among the weight classes and might suggest ARG common core representatives of the human gut resistome. This common core was composed by ARG related to bacterial resistance against tetracyclines, β-lactams, quinolones, aminoglycosides and sulphonamides. Although there are no Brazilian studies with which to compare the results, the data were consistent with other observations suggesting a common core of resistance markers in the gut clinical resistome of different populations from both South and North America, and Europe [8,9,29]. 

The frequency and diversity of the ARG were significantly higher within the obese group, with the exclusive presence of *tet*(M), *ere*A, *bla*Z, and *vgb* which confer resistance to tetracyclines, β-lactams and macrolides. In terms of ARG detection, overweight individuals behaved as an intermediate group, although one of the screened ARG was detected only within these individuals (*bla*_OXA-143_, related to β-lactam resistance in non-fermenter Gram-negative bacteria). 

Previous reports indicate that gut resistomes are more likely to correlate in terms of ARG abundance. This may be promoted by the widespread use of antimicrobial drugs in human and animal healthcare, such as β-lactams, and in food production, for example, tetracyclines as growth promoters in animals [9,29]. In Brazil, the use of chloramphenicol, β-lactams, tetracyclines, and sulfonamides have been banned from veterinary medicine since the late 1990s, and the use of other substances, such as arsenic, antimony, nitrofurantoin, and olaquindox, were banned at the beginning of 2000s. However, there are reports documenting how Brazil has managed the shift towards highly intensive livestock production systems, without legal requirement to obtain a veterinary prescription to use antibiotics in food animals [30,31] The use of these xenobiotics produced an environmental genetic pollution, as demonstrated by the presence of ARG in different environments [9]. It is also important to highlight xenobiotic intake as social behaviour or as chemotherapy, which have been linked to the modulation of bacterial populations in the human intestinal ecosystem [27,28]. In our current study the positive correlations between detection of at least five ARG and BMI > 25 (observed in overweight and obese groups), caloric intake, use of xenobiotics and artificial sweeteners was consistent with observations related to social behaviour and xenobiotic intake with regard to microbiota modulation, and highlighted the link between obesity, xenobiotic intake, and ARG yield in the gut resistome. 

Due to the variation in eating habits, it was not possible to establish correlations based on food consumption levels according to available nutritional guides [32]. Eating habits and social behaviours may interfere in the human intestinal microbiota inducing microbial metabolic stress that, together with xenobiotics associated with different foods, might play an important role as selective pressure for drug resistant bacteria [33,34]. 

Considering recent discussions on microbiota imbalance and shifts towards obesity, our observations are consistent with reports showing an increased population of *Bacteroides* and *Prevotella* (Bacteroidetes), *Fusobacterium*, *E. coli*, *Acinetobacter*, *Enterococcus*, and *Staphylococcus*, among others, in the gut microbiota of obese individuals [35]. Although in this study increased population was significant only for *E. coli* and *Enterococcus* towards obesity, a non-significant tendency in increased bacterial densities was observed for the other groups. Despite the well documented imbalance of Bacteroidetes, bacteria within this phylum have been associated with the presence of antibiotic resistance, especially tetracyclines and macrolides in the gut microbiota of adult individuals [36]. Furthermore, increased levels of Proteobacteria (such as Gram-negative enterobacteria like *Escherichia* in this study) are implicated in energy imbalance, as observed in individuals with metabolic and inflammation-associated disorders, such as obesity [37]. These findings might explain some observations in this study, such as the increased levels of *E. coli* in obese individuals, and the concomitant increased in ARG associated with Gram-negative rods, especially *Enterobacteriaceae*. Indeed, our results might suggest, by the ratio obtained between bacterial density in the different individual groups and the detection of ARG, that there was an association of ARG detection with Gram-negative rods, particularly in overweight and obese individuals. 

This study reinforced the hypothesis that obesity and its altered physiology may be implicated in gut microbiota imbalance. Furthermore, social and nutritional behaviour, and increased xenobiotic intake associated to obesity may play an important role in the gut microbiota, in addition to the antimicrobial resistance phenomenon. Individuals with a high BMI, high caloric intake and increased use of xenobiotics, such as artificial sweeteners and other systemic drugs, may harbour a greater and more diverse collection of ARG in their gut metagenome. This enhanced clinical resistome in obese individuals seems to be mainly associated with Gram-negative rods, and might be especially associated to the imbalance of Bacteroidetes and Proteobacteria. Further regional prospective studies are needed to better understand how different populations and habits may indirectly interfere with the human clinical resistome. This would help identify factors which may modulate the selective pressure in the gut and contribute to the bacterial drug-resistance phenomenon.

## Figures and Tables

**Table 1:** Correlation analysis between anthropometric and nutritional characteristics and habitual intake of xenobiotics by the participants and number of detected antimicrobial resistance genetic markers (ARG).

Characteristics	Odds ratio* (*Range*)
Body Mass Index (BMI) > 25	2.14 (0.75–6.07)
Mean daily calorie intake (≥ 8370 KJ or 2000 Kcal)	1.44 (0.51–4.07)
Habitual use of xenobiotics	1.93 (0.55–6.67)
Habitual use of artificial sweeteners	1.6 (0.40–6.64)

* OR, odds ratio, with 95% confidence interval: OR = 1.0, correlation absence; OR > 1.0, positive correlation; OR < 1.0, negative correlation.

**Table 2:** Ratios between the absolute numbers of detected antimicrobial resistance genetic markers (ARG) more likely to occur between the different bacterial groups and the relative frequency of each group, as determined by fluorescence in situ hybridization (FISH) in the faecal metagenome of the participants.

Participants	Bacterial Groups		
	Anaerobic Gran Negative Rods	Gram-Positive Cocci	Other Gram-Negative Bacilli
Eutrophic	0.60	4.20	8.63
Overweight	0.19	2.70	9.56
Obese	0.35	3.51	9.36

ARG considered for each group: anaerobic Gram-negative rods (*cfi*A, *cep*A); Gram-positive cocci (*aacA-aphD*, *mef*, *mph*A, *bla*Z, *ere*A, *ere*B, *vgb*); other Gram-negative bacilli (*amp*C, *bla*_CTX-M_, *bla*_TEM_, *bla*_SHV_, *bla*_OXA-2,_ *bla*_OXA-143_, *qnr*B, *qnr*S, *mex*Y, *tet*(A), *tet*(B), *tet*(E), *tet*(L), *tet*(M), *tet*(O), *tet*(Q), *sul*1, *sul*2).

## Supplementary Materials

The following are available online at https://www.mdpi.com/2073-4425/10/5/349/s1, Table S1: Primers used in this study for screening of antimicrobial resistance genetic markers (ARG), Table S2: Sequence of oligonucleotide probes used for fluorescence in situ hybridization, Table S3: Anthropometric and nutritional characteristics of the participants, Table S4: Declaration of habitual intake of xenobiotic, other than antimicrobial drugs, as reported by the participants.
