# Supplementary material for: Obesity, Xenobiotic Intake and Antimicrobial-Resistance Genes in the Human Gastrointestinal Tract: A Comparative Study of Eutrophic, Overweight and Obese Individuals
Source: Genes (Basel). 2019 May 7;10(5):349. doi: 10.3390/genes10050349 (PMC6562451; doi:10.3390/genes10050349)
Supplement: Supplementary file 1 [file genes-10-00349-s001.pdf]

SUPPLEMENTARY INFORMATION

Table S1. Primers used in this study for screening of antimicrobial resistance genetic markers (ARG).

Target	Primer sequence (5′ – 3′)	Amplicon (bp)	Reference
*bla*CTX-M	ATGTGCAGYACCAGTAAAG<br>GGTCACCAGAAGGAGC	562	*Antimicrob Agents Chemother*<br>2009; **53**: 465-475
*bla*KPC	ATGTCACTGTATCGCCGTCT<br>TTTTCAGAGCCTTACTGCCC	892	*Antimicrob Agents Chemother*<br>2009; **53**: 465-475
*bla*SHV	CTTTACTCGCCTTTATCGGC<br>TTACCGACCGGCATCTTTCC	982	*Antimicrob Agents Chemother*<br>2009; **53**: 465-475
*bla*TEM	GTGCGCGGAACCCCTATT<br>TTACCAATGCTTAATCAGTGAGGC	968	*Antimicrob Agents Chemother*<br>2009; **53**: 465-475
*bla*OXA-23	GATCGGATTGGAGAACCAGA<br>ATTTCTGACCGCATTTCCAT	501	*Int J Antimicrob Agents*<br>2006; **27**: 351-353
*bla*OXA-51	TAATGCTTTGATCGGCCTTG<br>TGGATTGCACTTCATCTTGG	353	*Int J Antimicrob Agents*<br>2006; **27**: 351-353
*bla*OXA-24	GGTTAGTTGGCCCCCTTAAA<br>AGTTGAGCGAAAAGGGGATT	246	*Int J Antimicrob Agents*<br>2006; **27**: 351-353
*bla*OXA-143	TGGCACTTTCAGCAGTTCCT<br>TAATCTTGAGGGGGCCAACC	149	*Int J Antimicrob Agents*<br>2010; **35**: 305
*bla*OXA-58	AAGTATTGGGGCTTGTGCTG<br>CCCCTCTGCGCTCTACATAC	599	*Int J Antimicrob Agents*<br>2006; **27**: 351-353
*bla*OXA-10	TCTTTCGAGTACGGCATTAGC<br>CCAATGATGCCCTCACTTTCC	760	*J Antimicrob Chemother*<br>2000; **46**: 703-711
*bla*OXA-2	GCCAAAGGCACGATAGTTG<br>GCGTCCGAGTTGACTGCCGG	701	*Antimicrob Agents Chemother*<br>2002; **46**: 3031-3034
*bla*Z	ACTTCAACACCTGCTGCTTTC<br>TGACCACTTTTATCAGCAACC	173	*Antimicrob Agents Chemother*<br>2000; **44**: 231-238
*cfx*A/*cfx*A2	CGTAGTTTTGAGTATAGCTTT<br>GATGTTGCCTATATATGTC	802	*J Antimicrob Chemother*<br>2003; **51**: 1293-1296
*amp*C	ATAACCACCCAGTCACGC<br>CAGTAGCGAGACTGCGCA	630	*Clin Infect Dis*<br>2002; **35**: 140-145
*bla*SPM-1	CCTACAATCTAACGGCGACC<br>TCGCCGTGTCCAGGTATAAC	649	*J Antimicrob Chemother*<br>2003; **52**: 699-702
*cfi*A	TCCATGCTTTTCCCTGTCGCAGTTAT<br>GGGCTATGGCTTTGAAGTGC	683	*J Med Microbiol*<br>2004; **53**: 413-419
tet(A)	GCTACATCCTGCTTGCCTTC<br>CATAGATCGCCGTGAAGAGG	210	*Mol Cell Probes*<br>2001; **15**: 209-215
tet(B)	TTGGTTAGGGGCAAGTTTTG<br>GTAATGGGCCAATAACACCG	659	*Mol Cell Probes*<br>2001; **15**: 209-215
tet(E)	AAACCACATCCTCCATACGC<br>AAA TAG GCC ACA ACC GTC AG	278	*Mol Cell Probes*<br>2001; **15**: 209-215
tet(K)	GTA GCG ACA ATA GGT AAT AGT<br>GTA GTG ACA ATA AAC CTC CTA	360	*J Clin Microbiol*<br>2003; **41**: 4089-4094
tet(L)	TCG TTA GCG TGC TGT CAT TC<br>GTA TCC CAC CAA TGT AGC CG	267	*Mol Cell Probes*<br>2001; **15**: 209-215
tet(M)	AGT GGA GCG ATT ACA GAA<br>CAT ATG TCC TGG GGT GTC TA	158	*J Clin Microbiol*<br>2003; **41**: 4089-4094
tet(O)	AGC GTC AAA GGG GAA TCA CTA TCC<br>CGG CGG GGT TGG CAA ATA	1723	*J Antimicrob Chemother*<br>2000; **45**: 763-770
tet(Q)	TTA TAC TTC CTC CGG CAT CG<br>ATC GGT TCG AGA ATG TCC AC	904	*Mol Cell Probes*<br>2001; **15**: 209-215
*mrs*A	TCC AAT CAT AGC ACA AAA TC<br>AAT TCC CTC TAT TTG GTG GT	163	*Jpn J Infect Dis*<br>2007; **60**: 183-187
*mec*A	GTA GAA ATG ACT GAA CGT CCG ATA A<br>CCA ATT CCA CAT TGT TTC GGT CTA A	310	*J Clin Microbiol*<br>2004; **42**: 4947-4955
mef	AGT ATC ATT AAT CAC TAG TGC	348	*Jpn J Infect Dis*

	TTC TTC TGG TAC AAA AGT GG		2007; **60**: 183-187
*ere*A	AAC ACC CTG AAC CCA AGG GAC G<br>CTT CAC ATC CGG ATT CGC TCG A	420	*Antimicrob Agents Chemother*<br>1996; **40**: 1817-1824
*ere*B	AGA AAT GGA GGT TCA TAC TTA CCA<br>CAT ATA AAT CAT CAC CAC CAA TGG CA	546	*Antimicrob Agents Chemother*<br>1996; **40**: 1817-1824
*mph*A	AAC TGT ACG CAC TTG C<br>GGT ACT CTT CGT TAC C	837	*Antimicrob Agents Chemother*<br>1996; **40**: 1817-1824
*erm*A	AAG CGG TAA ACC CCT CTG A<br>TTC GCA AAT CCC TTC TCA AC	190	*J Clin Microbiol*<br>2003; **41**: 4089-4094
*erm*B	CTA TCT GAT TGT TGA AGA AGG ATG AAA<br>GTT TAC TCT TGG TTT AGG ATG AAA	142	*J Antimicrob Chemother*<br>2000; **46**: 527-534
*erm*C	AAT CGT CAA TTC CTG CAT GT<br>TAA TCG TGG AAT ACG GGT TTG	299	*J Clin Microbiol*<br>2003; **41**: 4089-4094
*qnr*B	GAT CGT GAA AGC CAG AAA GG<br>ATG AGC AAC GAT GCC TGG TA	476	*Antimicrob Agents Chemother*<br>2009; **53**: 639-645
*qnr*S	GCA AGT TCA TTG AAC AGG GT<br>TCT AAA CCG TCG AGT TCG GCG	428	*Antimicrob Agents Chemother*<br>2009; **53**: 639-645
*sul*1	ATG GTG ACG GTG TTC GGC ATT CTG A<br>CTA GGC ATG ATC TAA CCC TCG GTC T	815	*J Antimicrob Chemother*<br>2003; **52**: 1022-1024
*sul*2	CCT GTT TCG TCC GAC ACA GA<br>GAA GCG CAG CCG CAA TTC AT	396	*Int J Antimicrob Agents*<br>2011; **37**: 230-234
*sul*3	GAG CAA GAT TTT TGG AAT CG<br>CAT CTG CAG CTA ACC TAG GGC TTT GGA	396	*J Antimicrob Chemother*<br>2003; **52**: 1022-1024
*nim*1	ATG TTC AGA GAA ATG CGG CGT AAG CG<br>GCT TCC TTG CCT GTC ATG TGC TC	458	*J Clin Microbiol*<br>1996; **34**: 2078-2084
*nim*2	ATG TTC AGA GAA ATG CGG CGT AAG CG<br>GCT TCC TTG CCT GTC ATG TGC TC	458	*J Clin Microbiol*<br>1996; **34**: 2078-2084
*nim*3	ATG TTC AGA GAA ATG CGG CGT AAG CG<br>GCT TCC TTG CCT GTC ATG TGC TC	458	*J Clin Microbiol*<br>1996; **34**: 2078-2084
*nim*4	ATG TTC AGA GAA ATG CGG CGT AAG CG<br>GCT TCC TTG CCT GTC ATG TGC TC	458	*J Clin Microbiol*<br>1996; **34**: 2078-2084
*aac*A-*aph*D	TAA TCC AAG AGC AAT AAG GGC<br>GCC ACA CTA TCA TAA CCA CTA	227	*J Clin Microbiol*<br>2003; **41**: 4089-4094
*vat*A	TGG TCC CGG AAC AAC ATT TAT<br>TCC ACC GAC AAT AGA ATA GGG	268	*J Clin Microbiol*<br>2003; **41**: 4089-4094
*vat*B	GCT GCG AAT TCA GTT GTT ACA<br>CTG ACC AAT CCC ACC ATT TTA	136	*J Clin Microbiol*<br>2003; **41**: 4089-4094
*vat*C	AAG GCC CCA ATC CAG AAG AA<br>TCA ACG TTC TTT GTC ACA ACC	467	*J Clin Microbiol*<br>2003; **41**: 4089-4094
vga	CCG AAC TGC TAT TAG CAG A<br>AAG TTC GTT TCT CTT TTC GA	470	*Antimicrob Agents Chemother*<br>1999; **43**: 1062-1066
vgb	ACT AAC CAA GAT ACA CAG GAC C<br>TTA TTG CTT GTC AGC CTT CC	734	*Antimicrob Agents Chemother*<br>1999; **43**: 1062-1066
*lin*A	GGT GGC TGG GGG GTA GAT GT<br>GCT TCT TTT GAA ATA CAT GG	323	*Antimicrob Agents Chemother*<br>1996; **40**: 1817-1824
*mrs*B	TAT GAT ATC CAT AAT AAT TAT CCA ATC<br>AAG TTTA TAT CAT GAA TAG ATT GTC CTG TT	334	*Antimicrob Agents Chemother*<br>1999; **43**: 1062-1066
*cep*A	TTT CTG CTA TGT CCT GCC C<br>ATC TTT CAC GAA GAC GGC	743	*Clinics*<br>2011; **66**: 543-547
*mex*B	GTG TTC GGC TCG CAG TAC TC<br>AAC CGT CGG GAT TGA CCT TG	244	*FEMS Microbiol Lett*<br>2005; **243**: 125-131
*mex*D	CGA GCG CTA TTC GCT GC<br>CGA GCG CTA TTC GCT GC	165	*BMC Microbiol*<br>2010; **10:217**
*mex*F	GGC AGT TGC ACG TCG A<br>CGC CTG GTC ACC GAG GAA GAG T	255	*Apmis*<br>2005; **113**: 187-196
*mex*Y	TAG TCC ATG GCT TGC GGG AAG C<br>CCG CTA CAA CGG CTA TCC CT	250	*FEMS Microbiol Lett*<br>2005; **243**: 125-131

Table S2. Sequence of oligonucleotide probes used for fluorescence in situ hybridization.

Probe	Target	Sequence (5′ – 3′)	Formamide	Reference
Bfrag998	*Bacteroides fragilis*	GTTTCCACATCATTCCACTG	30%	*Syst Appl Microbiol* 2003; **26**: 110-118
Bfra602	*Bacteroides fragilis* group	GAGCCGCAAACTTTCACAA	30%	*Appl Environ Microbiol* 1998; **64**: 3336
ECO1167	*Escherichia coli*	GCATAAGCGTCGCTGCCG	40%	*Syst Appl Microbiol* 1995; **18**: 113-122
Enc1259	*Enterococcus* spp.	GAAGTCGCGAGGCTAAGC	35%	*Syst Appl Microbiol* 2000; **23**: 563-572
FUS664	*Fusobacterium* sp.	CTTGTAGTTCCGCYTACCTC	40%	*Eur J Oral Sci* 2004; **112**: 33-41
PINT649	*Prevotella intermedia*	GCCGCCRCTGAASTCAGGCC	40%	*Microbiology* 2002; **148**: 1379-1387
PNG675	*Prevotella nigrescens*	TCCGCCTGCGCTGCGTGTA	40%	*Microbiology* 2002; **148**: 1379-1387
Pae997	*Pseudomonas* sp.	TCTGGAAAGTTCTCAGCA	35%	*Syst Appl Microbiol* 1995; **18**: 113-122
KO218	*Staphylococcus* sp.	GAAGCAAGCTTCTCG TCCG	20%	*J Microbiol Methods* 2005; **62**: 37-56
Str	*Streptococcus* spp.	CACTCTCCCCTTCTGCAC	30%	*Med Microbiol Immunol* 2000; **188**: 169-175
ACA652	*Acinetobacter* spp.	ATCCTCTCCCATACTCTA	35%	*Appl Environ Microbiol* 1994; **60**: 792-800

Table S3. Anthropometric and nutritional characteristics of the participants.

Characteristics (mean ± SD)	Groups of individuals			p < 0.05*
	**Eutrophic (n = 24)**	**Overweight (n = 24)**	**Obese (n = 24)**	
Gender (male/female)	05/19	12/12	09/15	
Average age (years ± SD)	37.91 ± 12	38.12 ± 13.38	42.79 ± 11.67	
Average Body Mass Index	22.81 ± 01.8	27.05 ± 01.3	36.92 ± 06.0	a,b,c
Abdominal circumference	88.05 ± 7.4	94.65 ± 5.5	114.98 ± 15.3	a,b,c
Waist circumference	80.95 ± 06.7	88.70 ± 4.5	111.99 ± 16.6	a,b,c
Hip circumference	99.60 ± 04.5	105.40 ± 4.30	117.45 ± 08.6	a,b,c
Waist-hip ratio	0.81± 0.55	0.84 ± 0.52	0.97 ± 0.12	a,b,c
Mean daily calorie intake (kcal)	1 891.58 ± 679	2 018.79 ± 845	2 191.33 ± 1,049	c
Mean daily carbohydrate intake (%)	53.60 ± 9.10	55.02 ± 7.78	57.43 ± 8.02	c
Mean daily lipids intake (%)	27.27 ± 8.79	29.87 ± 5.92	28.74 ± 5.60	-
Mean daily protein intake (%)	19.13 ± 4.30	15.11 ± 4.54	13.83 ± 3.27	-
Mean daily fibers intake (g)	23.75 ± 8.30	25.03 ± 13.30	30.49± 5.42	b,c

* Significant statistical analysis: *a*—comparison between eutrophic and overweight volunteers' values; *b*—comparison between overweight and obese volunteers' values; *c*—comparison between eutrophic and obese volunteers' values. SD = Standard Deviation

Table S4. Declaration of habitual intake of xenobiotic, other than antimicrobial drugs, as reported by the participants.

Xenobiotics		Habitual intake (%)		
		Eutrophic (n=24)	Overweight (n=24)	Obese (n= 24)
Antihypertensive		4.2	12.5	79.2
Antacids		0	0	8.4
Anti-inflammatories		0	4.2	8.4
Diuretic		0	0	4.2
Barbiturates		0	0	4.2
Antidepressant		0	0	4.2
Hormone Drugs (Tamoxifen)		0	4.2	0
Contraceptional		0	4.2	0
Nutritional Supplements	Whey protein	4.2	0	0
	Glucosamine Sulfate	4.2	0	0
	Chondroitin sulfate	4.2	0	0
	Others	8.4	0	4.2
Artificial sweeteners	Sucralose and Acesulfame K	8.4	8.4	16.6
	Stevioside and cyclamate-saccharin	0	4.2	0
	cyclamate-saccharin	4.2	8.4	25.0
	Others	0	0	0
Indeterminate		0	4.2	0
